# Mechanism of tobacco-sweet potato intercropping in suppressing *Ralstonia solanacearum* in flue-cured tobacco

**DOI:** 10.3389/fpls.2025.1688379

**Published:** 2025-11-17

**Authors:** Liqian Yang, Yang Liu, Shiping Guo, Tong Li, Qiuyue Nie, Yonghui Zhang, Shuhua Zeng, Fei Wang, Lei Liu

**Affiliations:** 1 Sichuan Agricultural University, Chengdu, China; 2 Luzhou City Tobacco Company of Sichuan Provincial Tobacco Company, Luzhou, China; 3 Sichuan Provincial Tobacco Company of China National Tobacco Corporation, Chengdu, China

**Keywords:** tobacco-sweet potato intercropping, *Ralstonia solanacearum*, phenolic acids, metabolomics, metagenomics

## Abstract

Tobacco bacterial wilt (*Ralstonia solanacearum*) is a fatal pathogen of tobacco, causing severe losses annually. Intercropping has been proposed as a sustainable strategy to mitigate soil-borne pathogens through rhizosphere interactions. However, the mechanisms by which tobacco-sweet potato intercropping specifically affects the microecological environment and suppresses *R. solanacearum* remain poorly understood. To investigate the effect of the TSP model on the soil-borne pathogen of bacterial wilt (*Ralstonia solanacearum*) in tobacco-growing soil, this study compared and analyzed the characteristics and differences in bacterial wilt incidence, *Ralstonia solanacearum* content, phenolic acid components, metabolome, and metagenome between (T) and (TSP) systems. The results showed that compared to the T treatment, the TSP treatment reduced the incidence of bacterial wilt in flue-cured tobacco and significantly decreased the abundance of *R. solanacearum* in the soil by 21.4%, while increasing the total phenolic acid content by 21.9%. The total phenolic content in the TSP soil was increased by 21.9% compared to T. Differentially abundant metabolites between TSP and T were primarily enriched in carbohydrate metabolic pathways, such as nucleotide sugar biosynthesis, fructose, and mannose metabolism. The content of substances such as rhamnose, D-allose, and mannitol in T-treated soil was 2.14–6.62 times higher than that in TSP-treated soil, with new tobacco alkaloids being up to 91.09 times higher. Compared to the T treatment, the TSP treatment significantly increased the relative abundances of Acidobacteriota, Chloroflexota, *Bradyrhizobium*, *Pseudolabrys*, and *Sphingomonas* by 64.08%, 18.86%, 23.55%, 21.80%, and 12.98%, respectively. The content of *Ralstonia solanacearum* in the soil was positively correlated with differential metabolites such as mannitol, rhamnose, and D-allose (r = 0.8), while negatively correlated with phenolic acids such as syringic acid, ferulic acid, caffeic acid, and gallic acid, as well as microorganisms such as Chloroflexota, Gemmatimonadota, Acidobacteriota, and *Sphingomonas*. In summary, TSP can regulate soil metabolites, phenolic acids, and beneficial microorganisms, forming a synergistic network to suppress the content of *Ralstonia solanacearum* and reduce the risk of tobacco bacterial wilt. This provides a theoretical basis for regulating soil microecology and enhancing crop disease resistance in intercropping systems.

## 1 Introduction

Tobacco bacterial wilt is a devastating bacterial disease caused by the soil-borne pathogen *Ralstonia solanacearum*. It invades the vascular system through root wounds, causing plants to wilt and die rapidly, especially in high-temperature and high-humidity environments (Liu et al., 2024). Current control measures for tobacco bacterial wilt primarily include selecting disease-resistant varieties, applying pesticides, regulating soil ecology, and implementing integrated agricultural pest management measures (Cai et al., 2018; Wei et al., 2022).

Intercropping refers to the practice of planting different crops in rows or strips on the same field during the same growing season (Zhu et al., 2024) and is a common integrated pest management strategy for crops. Under a reasonable intercropping pattern, the multiple cropping index and the utilization rate of field resources are improved (Qu and Wang, 2008), field ventilation and light conditions can also be enhanced, and the allelopathic effects of crops can be utilized to effectively suppress diseases (An et al., 2017). However, intercropping patterns may also have adverse effects, such as competition for growth resources, allelochemical inhibition, and increased difficulty in cultivation management and pest control. When two crops are intercropped, root exudates may recruit microbial communities that are detrimental to the growth of one of the crops (Zhou et al., 2023), and the accumulation of allelochemicals may cause toxic effects.

Long-term continuous cropping of tobacco can lead to deterioration of soil physical and chemical properties (Ma et al., 2021), an imbalance of the microbial community structure (Teng et al., 2020), and exacerbation of soil-borne diseases such as tobacco bacterial wilt (Wang et al., 2008). Sweet potatoes are an important crop that can be used as food, vegetables, and bioenergy (Wang et al., 2021). They contain components, such as sugars and polyphenols, which have antioxidant and antibacterial effects (Sun et al., 2018). The tobacco-sweet potato intercropping system is a highly beneficial integrated model that has been widely adopted. Long-term use of tobacco-sweet potato intercropping or rotation can lead to an increase in soil fungal ratios and a decrease in microbial numbers and activity (Yang et al., 2013). The thienyl-containing fungicidal or bacteriostatic substances, such as BBT, secreted by the root systems of tobacco-sweet potato intercropped crops can exert fungicidal effects, keeping the population of *Ralstonia solanacearum* at a low level (Zhou et al., 2020), thereby effectively reducing the incidence of soil-borne diseases such as tobacco bacterial wilt (Shi et al., 2011). Tobacco-potato intercropping increases soil microbial community richness, improves microbial community structure, and enhances the proportion of dominant microbial groups such as Actinobacteria and Acidobacteria (Du et al., 2025), while significantly increasing the number of microorganisms with antagonistic effects against pathogens (Wang et al., 2024).

During intercropping, root and soil metabolites are the basis for interactions between different crops and also reflect the outcomes of crop interactions. Soil metabolites primarily include plant root exudates, microbial metabolic activities, and the decomposition of organic matter in the soil (Shi et al., 2024). Root exudates refer to the totality of various organic substances released by plant roots into the rhizosphere environment, whose components include sugars, organic acids, phenolic acids, etc (Fang and Yue, 2024). Studies have found that phenolic acids can significantly influence soil microbial biomass, diversity, and community structure, and selectively promote the enrichment of specific microbial species (Qu and Wang, 2008) The accumulation of coumaric acid inhibits the enrichment of *Bacillus* but promotes the proliferation of *Streptomyces* (Chen et al., 2023). Glucose, as a carbon source, can simultaneously enhance both the activity and abundance of soil microorganisms (Nogales et al., 2011).No studies have been reported on the effects of tobacco-sweet potato intercropping on phenolic acids and metabolomics in tobacco-growing soils. Clearly, the tobacco-sweet potato intercropping system has significant impacts on crops, soil, and microorganisms, with their complex interrelationships necessitating further investigation using multi-omics analytical techniques.

To investigate the effects of the tobacco-sweet potato intercropping system on *Ralstonia solanacearum* (the pathogen causing tobacco bacterial wilt) in tobacco-planted soil and its underlying mechanisms, this study utilized metabolomics and metagenomics technologies to analyze the effects of the tobacco-sweet potato intercropping system on *Ralstonia solanacearum*, phenolic acid content, metabolites, and the functional diversity of the rhizosphere microbial community in tobacco-planted soil as well as to explore the relationships among these factors.

## 2 Materials and methods

### 2.1 Experimental site selection and design

Test site: Located in Dazhai Township, Gulin County, Luzhou City, Sichuan Province, China, at 105.43°E, 28.87°N, with an average altitude of 1,021 m, it is in a subtropical plateau climate zone. The soil at the experimental site is sandy loam, with a pH of 5.71, alkali-hydrolyzable nitrogen of 141 mg/kg, available phosphorus of 40.77 mg/kg, available potassium of 148.9 mg/kg, and an organic matter content of 41.67 g/kg.

Crop Varieties: The tobacco variety was Zhongchuan 208, and the sweet potato variety was Yusu 303.

**Figure 1:**
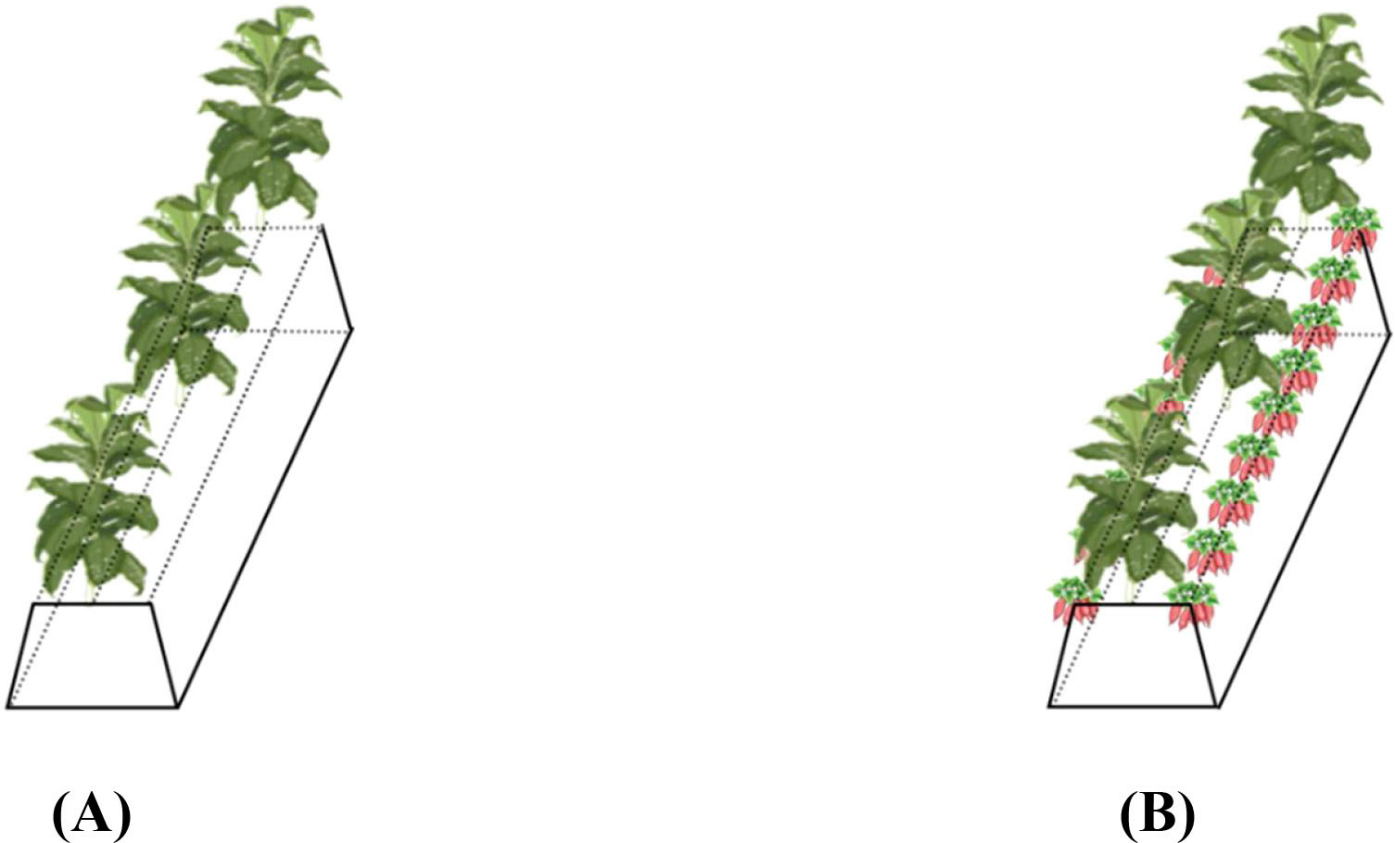
**(A)** Tobacco monoculture. **(B)** Tobacco-sweet potato intercropping.

Field experiments: Tobacco monoculture (T) was planted in single rows on ridges, as shown in **Figure 1A**, with a ridge base width of 50 cm, ridge top width of 40 cm, and ridge height of 30 cm; the plant spacing was 0.5 × 1.2 m; tobacco was transplanted on April 15. Tobacco-sweet potato intercropping (TSP) was arranged as shown in **Figure 1B**. The intercropped sweet potatoes were transplanted on June 3, with one row of sweet potatoes planted on each side of the tobacco ridge top and a plant spacing of 0.25 m (105,600 plants/hm²). Crops were harvested by October 8.

Basal fertilizer: Before ridge formation, 750 kg/hm² of tobacco-specific compound fertilizer 300 kg/hm² of oil cake organic fertilizer were applied in bands. Topdressing: 75 kg/hm² and 300 kg/hm² of tobacco-specific compound fertilizer were applied at 10 and 30 days after transplanting, respectively. No additional fertilizer was applied after sweet potato transplanting.

### 2.2 Sample collection and analysis

Thirty days after the TSP treatment, soil samples were collected from the root zones of both treatments (specifically from the 5–10 cm soil layer in the T treatment, and from the root interaction zones between the two crops in the TSP treatment) at a depth of 5–10 cm. Sampling was conducted using the five-point sampling method, with soil samples collected from the root distribution areas of tobacco and sweet potato. After removing roots and visible stones, the samples were mixed and sieved through a 2 mm sieve. Six replicate samples (6 g each) were collected from each treatment. A portion of each soil sample was air-dried and packaged for measuring soil pathogen content and soil phenolic acid content; the remaining portion was stored in cryogenic tubes using liquid nitrogen for soil metabolomics and soil microbial metagenomic sequencing analysis.

#### 2.2.1 Determination of soil pathogen content

Total DNA was extracted using the Omega Soil DNA Kit (D5625), with 80 μL of elution buffer recovered. DNA quality was confirmed via 1.5% agarose gel electrophoresis and NanoDrop detection (OD260/280 = 1.63–1.76, concentration 22.2–35.8 ng/μL). For *Ralstonia solanacearum* (tobacco bacterial wilt pathogen), the fliC gene primers were used (Rsol_fliC-F: GAACGCCAACGGTGCGAACT; Rsol_fliC-R: GGCGGCCTTCAGGGAGGTC). Plasmid standards were constructed via cloning and sequencing (Rsol_fliC: 400 bp, plasmid copy number 8.73×10^10^ copies/μL, and a 10-fold serial dilution was used to prepare the standard curve (R² > 0.99). qPCR reactions were performed using SGExcel FastSYBR Mixture (Sangon) on the LightCycler 480 II system. The 20 μL reaction mixture contained 1× SYBR Mix, 0.2 μM primers, and 1 μL template DNA (Ralstonia template diluted 10×). The program was as follows: 95 °C pre-denaturation for 4 min; 10 cycles (95 °C for 10 s, 67 °C → 57 °C for 20 s per cycle, -1 °C per cycle); 35 cycles (95 °C for 10 s, 57 °C for 20 s, 72 °C for 30 s). Pathogen content was expressed as log_10_(gene copy number/g dry soil). For specific measurement methods, refer to the study by Li Fengwei et al (Li et al., 2023).

#### 2.2.2 Determination of soil phenolic acids

The extraction of soil phenolic acids was performed according to the method described by Tian Geilin (Tian et al., 2015) et al. HPLC was used to determine phenolic acids such as p-hydroxybenzoic acid, vanillic acid, cinnamic acid, p-coumaric acid, ferulic acid, and benzoic acid in soil. The sample was filtered through a 0.22 μm microporous membrane before injection, with an injection volume of 10 μL, a flow rate of 0.8 mL·min-1, a detection wavelength of 280 nm, mobile phase A as acetonitrile, mobile phase B as 0.2% phosphoric acid solution (A:B = 24:76), and a Diamonsil C18 chromatographic separation column. Column temperature was maintained at 30 °C. Qualitative analysis was performed based on retention times in the HPLC chromatogram, and quantitative analysis of each phenolic acid compound was conducted using the peak area external standard method.

#### 2.2.3 Soil metabolomics analysis

Six biological replicates per treatment were processed as follows: Samples were retrieved from the -80 °C freezer, thawed on ice, and precisely 250 mg (± 5 mg) of each sample was weighed into centrifuge tubes with recorded weights; after adding 500 μL of -20 °C pre-cooled 70% methanol aqueous internal standard extraction solution, samples were vortexed for 3 min (with steel beads added for an additional 3 min vortexing if incomplete dispersion was observed), sonicated in an ice-water bath for 10 min, vortexed again for 1 min, incubated at -20 °C for 30 min, centrifuged at 4 °C and 12,000 r/min for 10 min, and finally the entire supernatant was aspirated and filtered through a 0.22 μm PTFE membrane for instrumental analysis under the following. The raw data from mass spectrometry were calibrated, and the peaks screened after calibration were subjected to metabolite identification by searching against a laboratory-built database, integrated public databases, predictive databases, and the metDNA method. T3 chromatographic conditions: Waters ACQUITY Premier HSS T3 Column (1.8 µm, 2.1 mm × 100 mm) with mobile phase A (0.1% formic acid in water) and B (0.1% formic acid in acetonitrile), column temperature 40 °C, flow rate 0.4 mL/min, and injection volume 4 μL, using a TripleTOF 6600+ high-resolution mass spectrometer (SCIEX, Foster City, CA, USA) coupled with an LC-30A ultra-performance liquid chromatography system (Shimadzu, Japan).

#### 2.2.4 Soil DNA sample detection, library construction, library quality control, and sequencing

Agarose gel electrophoresis (AGE) was used to analyze DNA purity and integrity; Qubit was used for precise quantification of DNA concentration. Qualified DNA samples were randomly fragmented into fragments of approximately 300 bp using a Covaris ultrasonic disruptor, followed by end repair, A-tailing, addition of sequencing adapters, purification, PCR amplification, and other steps to complete the library preparation process. After library construction, the library was initially quantified using Qubit 2.0, diluted to 2 ng/μl, and then the insert size was detected using Agilent 2100. The effective concentration of the library was accurately quantified using Q-PCR (effective library concentration >3 nM) to ensure library quality. Different libraries were pooled according to their effective concentration and target offline data volume requirements and then sequenced using Illumina PE150 (Han et al., 2025).

### 2.3 Statistical analysis

Soil phenolic acid data were assessed using one-way analysis of variance (ANOVA) with Duncan’s test in SPSS 25.0 (IBM SPSS Inc., USA). Differences were considered statistically significant at p < 0.05. LEfSe analysis was performed to assess functional differences between groups based on functional abundance. Pearson correlation tests were used to evaluate the correlations between soil metabolites, pathogens, phenolic acids, and microorganisms.

## 3 Results

### 3.1 Tobacco-sweet potato intercropping significantly reduces the amount of tobacco bacterial wilt

**Figure 2:**
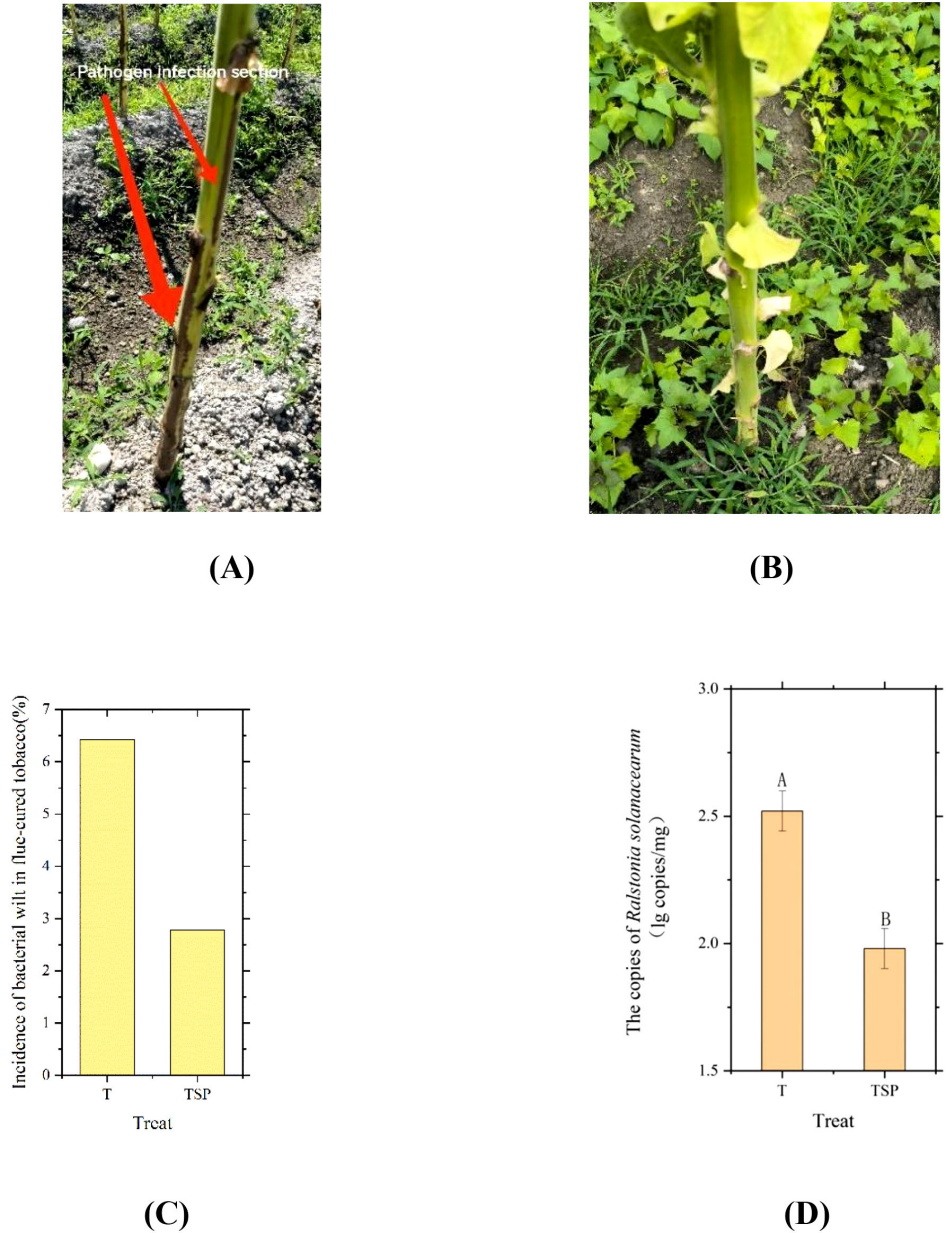
Disease incidence and soil pathogen content in T and TSP treatments. **(A)** Photograph of flue-cured tobacco plants infected with bacterial wilt. **(B)** Photograph of flue-cured tobacco plants not infected with bacterial wilt. **(C)** Incidence of bacterial wilt in flue-cured tobacco under T and TSP treatments on July 4 (corresponding to the omics sampling time point). **(D)** Quantitative PCR measurement of *Ralstonia solanacearum content* in soil under T and TSP treatments Data are means of six replicates.Different letters indicate significant differences at p < 0.01 level. T-test.

As presented in **Figures 2A** and **2B**, these depict a flue-cured tobacco plant in the field presenting with bacterial wilt symptoms and an asymptomatic plant, respectively. Statistical analysis of bacterial wilt incidence in flue-cured tobacco (**Figure 2C**) indicated that the disease incidence rate was higher in the (T) treatment than in the (TSP) treatment. As shown in **Figure 2D**, the copy numbers of *Ralstonia solanacearumin* T and TSP treated soils were 2.52 and 1.98 (lg copies/mg), respectively. The pathogen content in TSP was 21.4% lower than that in T, with a highly significant difference between the two treatments (p< 0.01). These results demonstrate that the TSP system effectively reduces both the incidence of tobacco bacterial wilt and the content of *R. solanacearumin s*oil.

### 3.2 Analysis of phenolic acid content in soil between treatments

Phenolic acids exert a bidirectional regulatory effect (inhibitory or promotional) on soil microorganisms, significantly influencing soil ecological functions. TSP treatment had a significant impact on the content of phenolic acid compounds in soil, with notable increases or decreases observed in the levels of various phenolic acids (**Table 1**). The content of key phenolic compounds in TSP, including epicatechin, vanillic acid, syringic acid, kaempferol, catechol, gallic acid, p-hydroxybenzoic acid, sinapic acid, and syringaldehyde, increased by 61.1%, 46.0%, 33.07%, 33.46%, 32.88%, 32.61%, 20.83%, 10.53% and 6.64%, respectively, compared with those under T treatment. Conversely, the levels of o-coumaric acid, catechin, chlorogenic acid, rutin, and oleanolic acid decreased by 37.5%, 37.4%, 28.57%, 20% and 17.89%, respectively. The total content of soil phenolic acids in the TSP condition was significantly higher than under sole tobacco cropping, with an increase of 21.9%. The TSP intercropping system significantly increased the total phenolic content in soil and altered the proportional relationship of phenolic acid components, which may have an impact on the *Ralstonia solanacearum* pathogen in tobacco.

**Table 1:** Phenolic acid content in single-crop and intercropped soils (ng/g).

Number	Name	T	TSP	Significance
1	Syringic acid	697.99	928.85	**
2	Ferulic acid	173.25	178.20	**
3	Quinic acid	0.43	0.40	*
4	Caffeic acid	8.92	9.24	**
5	Gallic acid	3.68	4.88	**
6	Phthalic acid	552.19	571.64	**
7	Protocatechuic acid	97.77	101.75	**
8	p-Coumaric acid	843.07	828.46	**
9	o-Coumaric acid	0.64	0.40	**
10	Catechol	0.73	0.97	**
11	Benzoic acid	485.60	487.87	ns
12	p-Hydroxybenzoic acid	1346.09	1626.52	**
13	Protocatechualdehyde	100.80	105.80	**
14	Syringaldehyde	144.93	154.55	**
15	Sinapic acid	11.40	12.60	**
16	Kaempferol	2.63	3.51	**
17	Epicatechin	0.72	1.16	**
18	Catechin	1.23	0.77	**
19	Chlorogenic acid	0.14	0.10	**
20	Rutin	0.05	0.04	**
21	Vanillic acid	1940.49	2832.74	**
22	Oleanolic acid	8.05	6.61	**
23	Vanillin	174.45	187.39	**
24	Total phenolic acid content​	6595.29	8044.48	**

* indicates a significant difference between groups T and TSP (*p < 0.05, **p < 0.01, t-test). Data are means of six replicates.

### 3.3 Metabolome analysis of the soil between treatments

**Figure 3:**
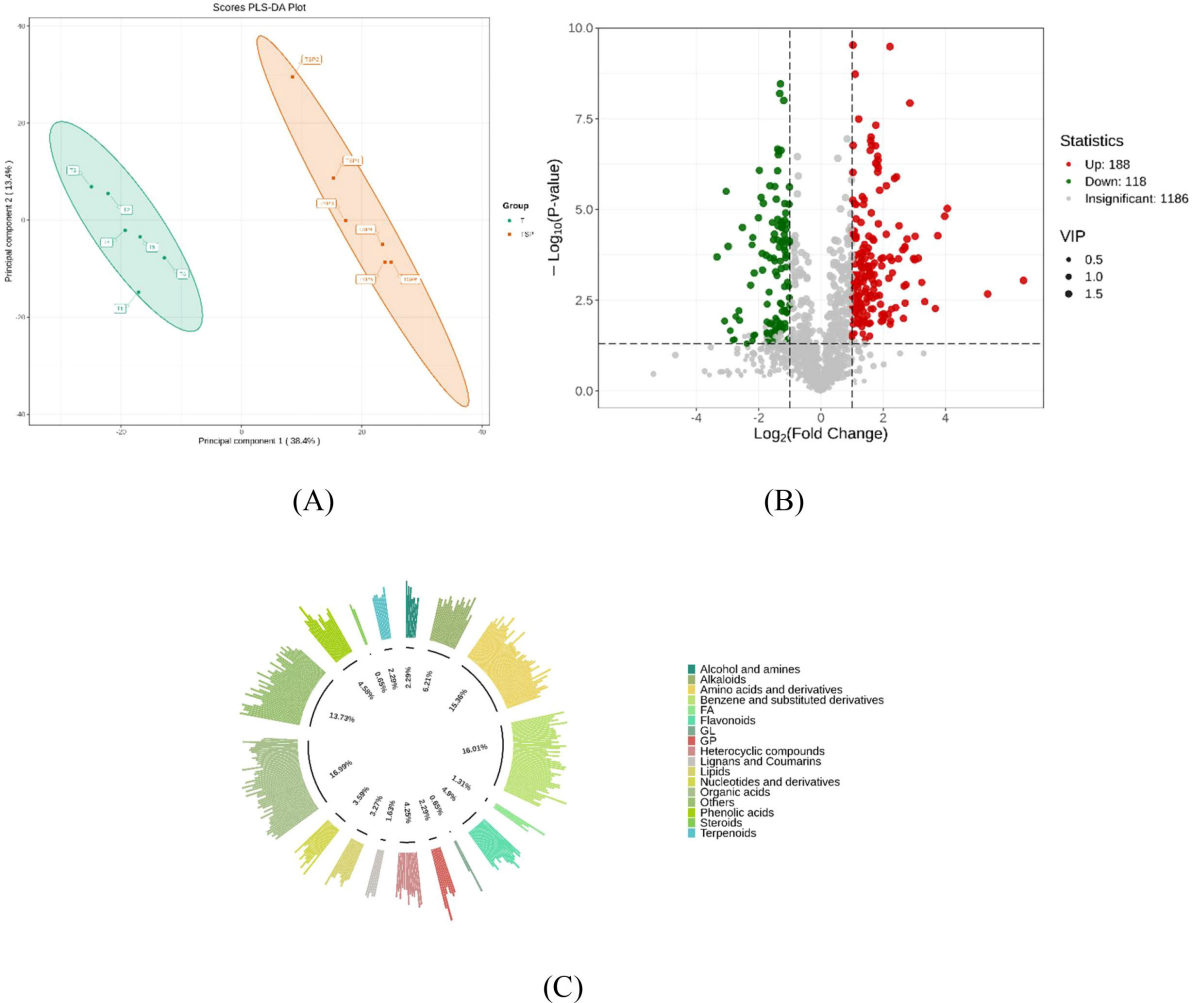
Analysis of differential metabolite abundance under T and TSP conditions. **(A)** PLS-DA analysis of soil metabolites in tobacco monoculture vs. tobacco-sweet potato intercropping**. (B)**: Volcanic plot of metabolic differences between tobacco monoculture and tobacco-sweet potato intercropping. (VIP > 1, |log_2_FC| > 1, p < 0.05). Red dots represent upregulation, green dots represent downregulation, and gray dots indicate no significant difference. **(C)** Proportion of different metabolites between tobacco monoculture and tobacco-sweet potato intercropping. Data are means of six replicates.

Partial Least Squares Discriminant Analysis (PLS-DA) analysis was performed on metabolites in TSP and T soils, with results shown in **Figure 3A**. The results indicate that the TSP and T sample groups exhibit clear categorical divisions and significant differences. Differential analysis (**Figure 3B**) revealed 306 significantly different metabolites (VIP > 1, |log_2_FC| > 1, p < 0.05) between the two groups. Among these, 188 substances were significantly upregulated and 118 were significantly downregulated under T treatment. The categories of differentially abundant metabolites between T and TSP are shown in **Figure 3C** and included organic acids (16.99%), benzene and its derivatives (16.01%), amino acids and their derivatives (15.36%).

The main differentially abundant metabolites between T and TSP (**Table 2**) included quinolinic acid, nicotine, mannitol, rhamnose, and D-allose.

**Table 2:** Main differences in soil metabolites between tobacco monoculture (T) and tobacco-sweet potato intercropping (TSP).

Index	Compounds	VIP	P-value	Fold_Change	Type
MEDP0247	Nicotinuric acid	1.72	0.00	8.73	up
MEDN0541	DL-3,4-Dihydroxyphenyl glycol	1.54	0.00	2.03	up
MEDL02220	Nicotine	1.68	0.00	6.62	up
MW0148779	dTDP-beta-L-4-epi-vancosamine	1.51	0.00	9.45	up
MEDN1011	Mannitol	1.61	0.00	3.29	up
MEDN0232	Rhamnose	1.74	0.00	2.14	up
MEDL00581	D-Allose	1.59	0.01	2.15	up
MW0114960	N-acetyl-alpha-D-glucosamine 1-phosphate	1.72	0.00	3.56	up
MW0105414	Acetoacetic acid	1.63	0.00	2.00	up
MW0106105	Carbamic acid	1.69	0.00	2.04	up
MEDN1161	Quinolinic acid	1.52	0.00	-1.52	down
MEDP0232	N-Acetyl-D-glucosamine	1.72	0.00	-1.16	down

Data are means of six replicates.

Mannitol, rhamnose, and other sugars serve as carbon/energy sources for *Ralstonia solanacearum* in soil or host tissues, supporting its survival and exerting significant impacts on soil-borne pathogens (Wang et al., 2019). In T-treated soils, the concentrations of substances including mannitol, rhamnose, and D-allose were markedly upregulated compared to TSP-treated soils. This phenomenon may stem from higher *Ralstonia solanacearum* levels in in T-treated soils, which activate plant root systems to synthesize and secrete more osmoregulatory sugars.

### 3.4 KEGG pathway enrichment analysis of differential metabolites in tobacco-sweet potato intercropped soil

**Figure 4:**
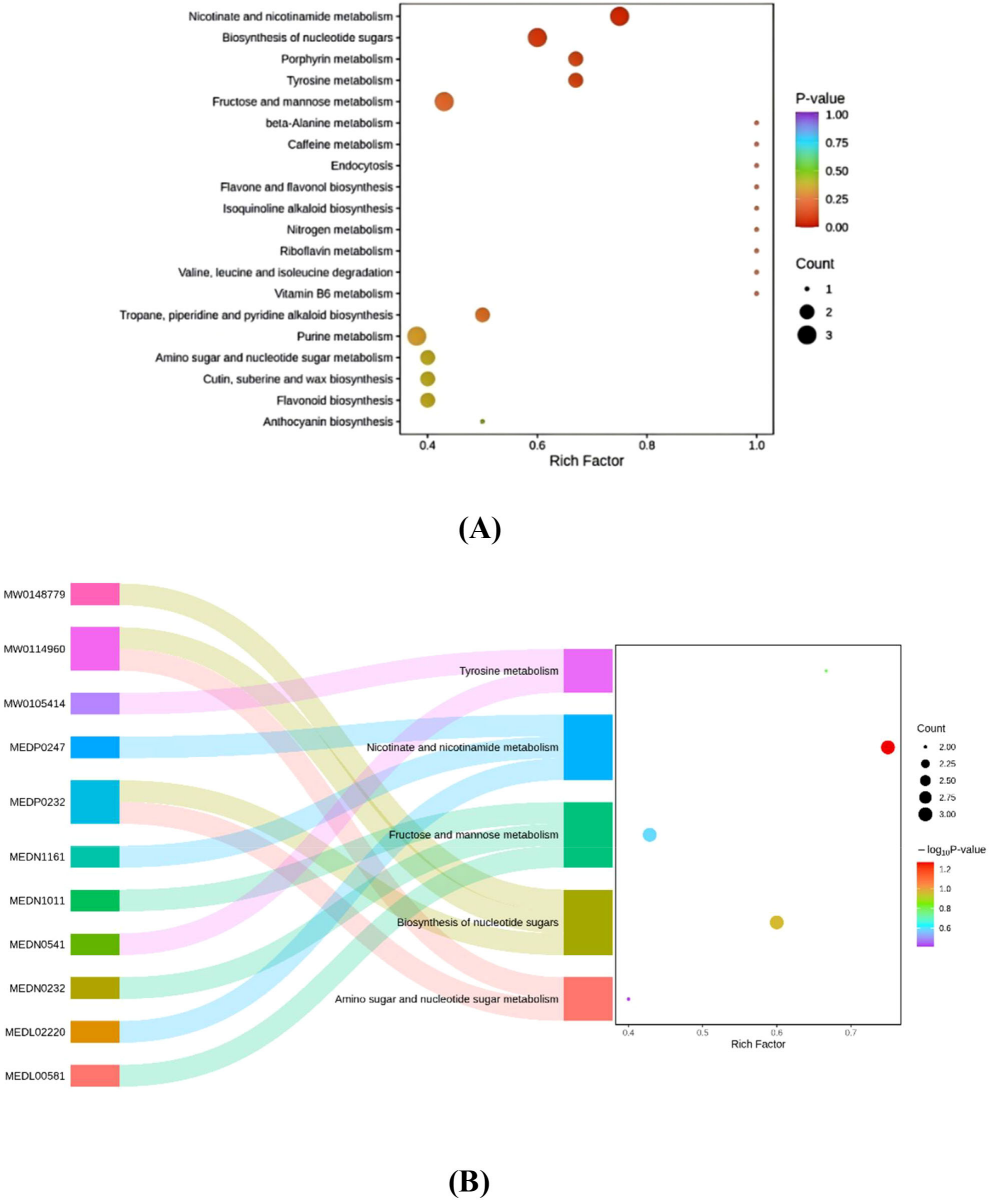
**(A)** Metabolic pathway diagram showing differences in metabolite accumulation between tobacco monoculture and tobacco-sweet potato intercropping**. (B)** Sankey diagram showing differences in metabolites between tobacco monoculture and tobacco-sweet potato intercropping. Data are means of six replicates.

KEGG enrichment bubble plots and Sankey diagrams (**Figures 4A, B**) demonstrate that differential metabolites—including rhamnose, mannose, D-allose, carbamate, nicotine, and nicotinylglycine—between monocropped flue-cured tobacco and tobacco-sweet potato intercropping systems are primarily enriched in nicotinate and nicotinamide metabolism, nucleotide sugar biosynthesis, tyrosine metabolism, and fructose and mannose metabolism pathways. Among these, nicotinate and nicotinamide metabolism, nucleotide sugar biosynthesis, tyrosine metabolism, and fructose and mannose metabolism represent significantly enriched pathways, each featuring 2–3 significantly differential metabolites. In summary, these substances and pathways are all related to plant disease resistance and stress tolerance.

### 3.5 Soil metagenomic analysis of the tobacco-sweet potato intercropping system

#### 3.5.1 Soil microbial α diversity in tobacco monoculture and intercropping

The α-diversity of microbial communities differed significantly between T and TSP conditions (**Table 3**). Compared with TSP, the observed species, Shannon, Chao1, and ACE indices in T were significantly increased by 14%, 2.6%, 14%, and 14%, respectively, indicating higher species richness and diversity in T. No significant differences were detected in Simpson and Goods_coverage indices, indicating that both treatments did not significantly alter the evenness of species distribution within the microbial community; The sequencing depth was relatively sufficient across all samples, with comparable and adequate sampling coverage, demonstrating similar representativeness of microbial diversity between the two systems. These results demonstrate that the TSP treatment reduced soil microbial richness and species diversity, which may be associated with the root activities of tobacco and sweet potato.

**Table 3:** Soil microbial α diversity in tobacco monoculture and intercropping.

Alpha_diversity	T	TSP	Significance
observed_species	12217.33	10718.17	**
Shannon	3.56	3.47	**
Simpson	0.76	0.76	ns
Chao1	12217.36	10718.17	**
ACE	12217.43	10718.19	**
Goods_coverage	1	1	ns

* indicates a significant difference between groups T and TSP (*p < 0.05, ** p < 0.01, t-test). Data are means of six replicates.

#### 3.5.2 Effects of tobacco-sweet potato intercropping on soil microbial community structure

**Figure 5:**
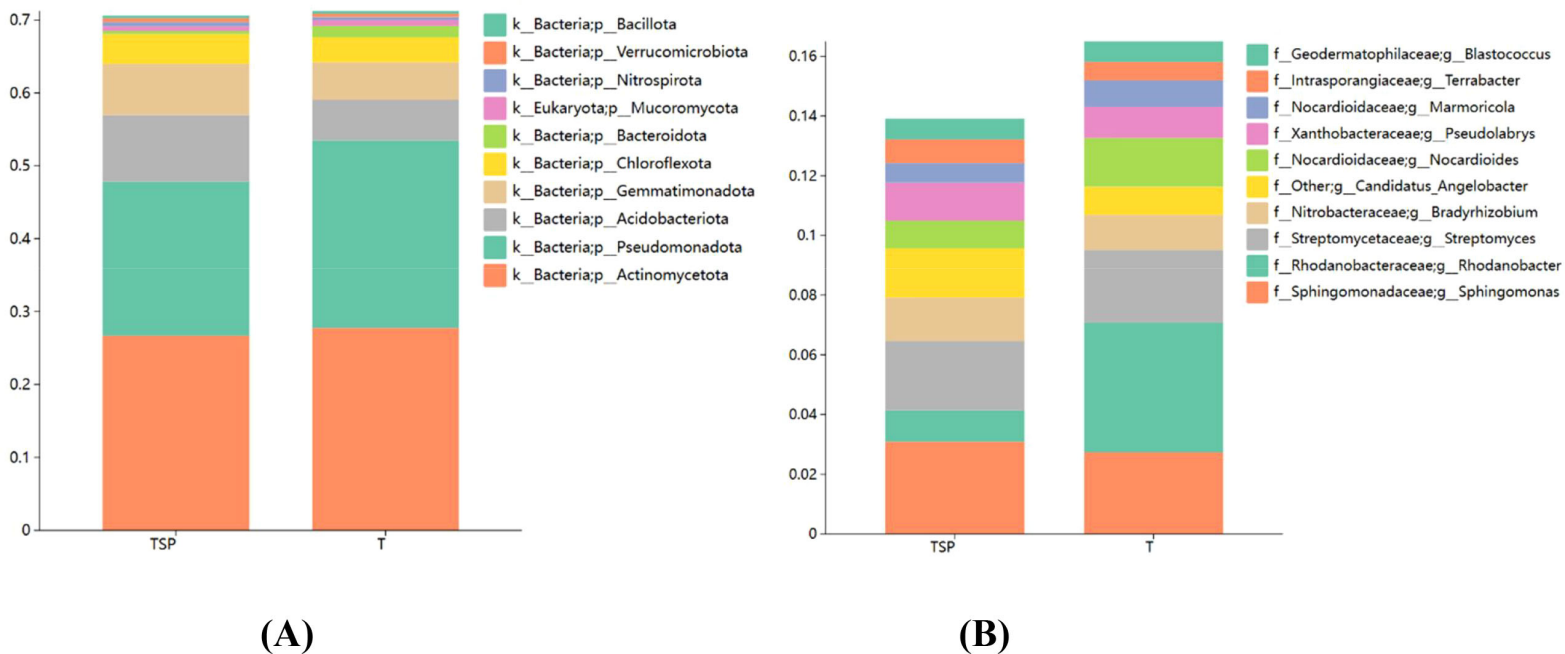
Bacterial species abundance in soils. **(A)** Top 10 species abundance at the species level**. (B)** Top 10 species abundance at the genus level. Data are means of six replicates.

As shown in **Figure 5A**, the top 10 dominant bacterial phyla in the relative abundance of rhizosphere soil in each treatment were Actinomycetota, Pseudomonadota, Acidobacteriota, Gemmatimonadota, Chloroflexota, Bacteroidota, Mucoromycota, Nitrospirota, Verrucomicrobiota, Bacillota, with a total relative abundance of 70%. Compared to the T treatment, the TSP treatment significantly increased the relative abundances of microorganisms such as Acidobacteriota, Gemmatimonadota, Nitrospirota, Chloroflexota, and Verrucomicrobiota by 64.08%, 36.38%, 36.13%, 18.86%, and 15.41%, respectively; while significantly decreasing the relative abundances of Bacteroidota, Pseudomonadota, and Mucoromycota by 71.97%, 17.93%, and 17%, respectively.

As shown in **Figure 5B**, the top 10 dominant bacterial genera in terms of relative abundance in the rhizosphere soil of each treatment were *Sphingomonas*, *Rhodanobacter*, *Streptomyces*, *Bradyrhizobium*, *Candidatus Angelobacter*, *Nocardioides*, *Pseudolabrys*, *Marmoricola*, *Terrabacter*, and *Blastococcus*. Compared to the T treatment, the TSP treatment significantly increased the relative abundances of microorganisms including *Candidatus_Angelobacter*, *Terrabacter*, *Bradyrhizobium*, *Pseudolabrys*, and *Sphingomonas* by 74.50%, 27.13%, 23.55%, 21.80%, and 12.98%, respectively; while significantly decreasing the relative abundances of *Rhodanobacter*, *Nocardioides*, and *Marmoricola* by 75.79%, 43.29%, and 25.2%, respectively. These results indicate that in intercropping systems, plants can recruit beneficial bacterial communities, enhancing the health of tobacco-growing soils and reducing the incidence of tobacco diseases.

#### 3.5.3 Species-level differences in the intercropping system

**Figure 6:**
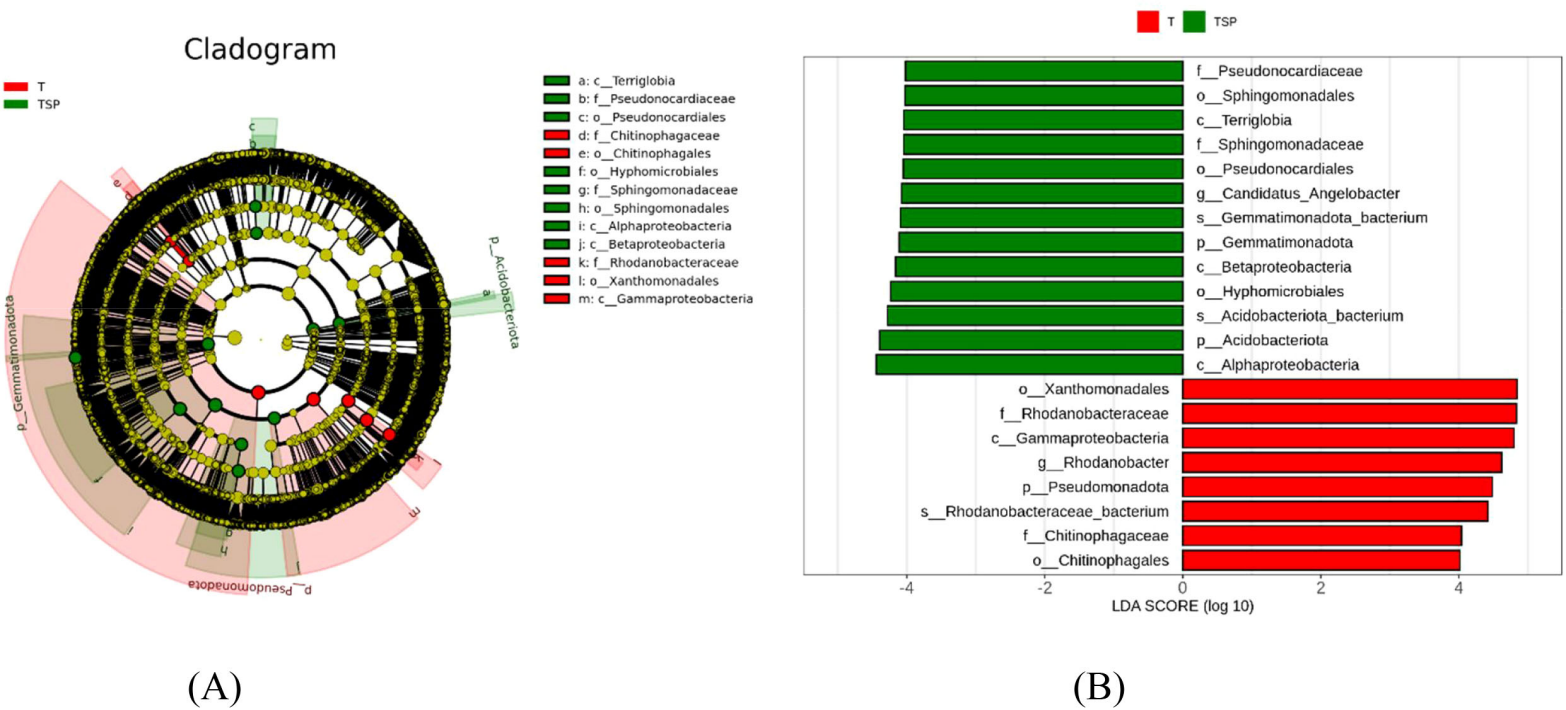
**(A)** Evolutionary branch diagram of species differing between T and TSP**. (B)** LDA bar chart of species differing between T and TSP. Data are means of six replicates.

The Linear Discriminant Analysis(LDA) score threshold indicated that there are significant differences in soil microbial species under different treatments. Linear discriminant analysis (LEfSe) of the T vs. TSP group (**Figures 6A, B**) showed 21 biomarkers (LDA > 4). The T group contained eight major biomarkers, among which the genus-level difference in *Candidatus_Angelobacter* had the most significant effect. The TSP group contained 13 biomarkers, among which the genus-level difference in *Rhodanobacter* had the most significant effect.

#### 3.5.4 KEGG functional annotation analysis of the intercropping system

**Figure 7:**
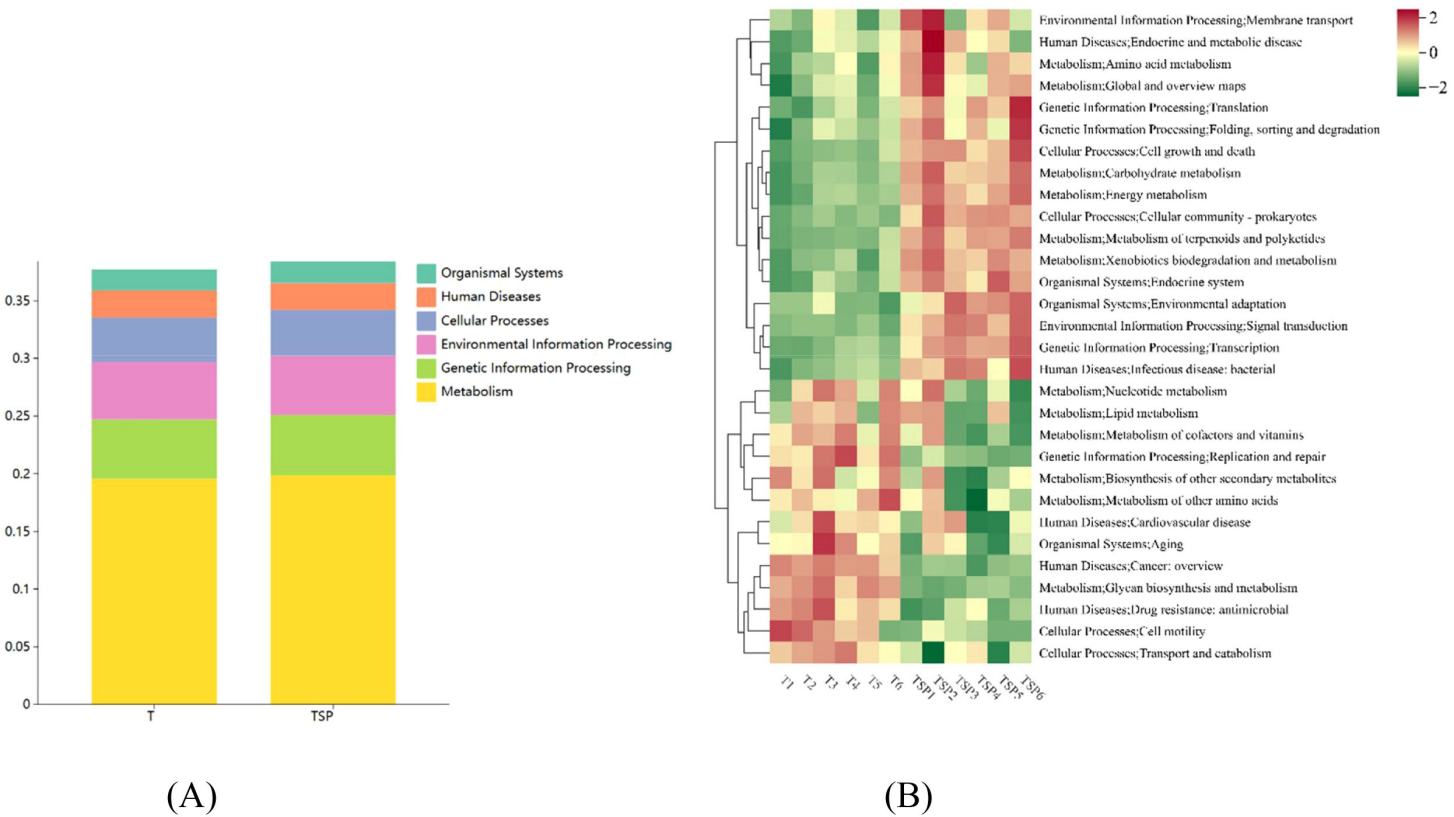
KEGG analysis of unigenes between the TSP and T conditions. **(A)** Level 1 KEGG functional annotation of TSP and T**. (B)** TSP and T’s tertiary KEGG functional annotation. Data are means of six replicates.

Mapping annotated unigenes to the KEGG database yielded six functional categories (Level 1 KEGG pathways) (**Figure 7A**) and 30 Level 3 KEGG pathways (**Figure 7B**).

KEGG pathway enrichment analysis reflects the adaptive evolution of soil microorganisms to the diversified cropping environment under intercropping treatments. This is primarily manifested in metabolic enrichment, accounting for up to 19% of the enriched terms. Differential metabolite analysis indicated that TSP increases the content of amino acids and complex carbohydrates in the soil. Under TSP treatment, terms related to environmental information processing and membrane transport accounted for 5%, showing a significant upregulation compared to T treatment, indicating that intercropping measures triggered microbial responses to environmental changes. Of enriched terms, 3.8% were related to cellular processes, indicating that TSP intercropping triggers extensive cell growth and death in rhizosphere soil, initiating the restructuring of microbial community structure and function.

### 3.6 Correlation analysis between omics data

**Figure 8:**
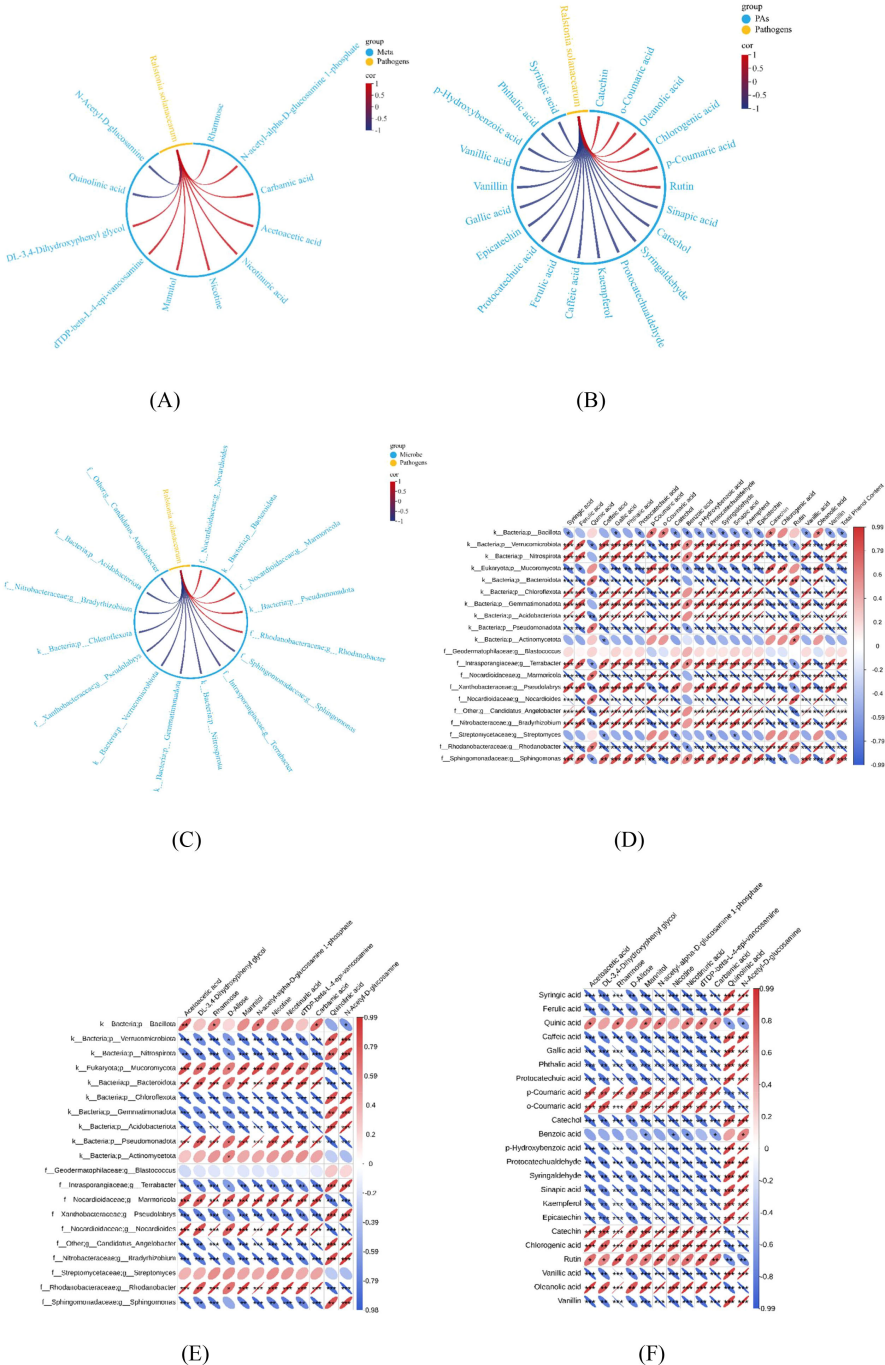
Correlation analysis. **(A)** Correlation analysis between soil bacterial wilt content and differential metabolites**. (B)** Correlation analysis between soil bacterial wilt pathogens and phenolic acid content**. (C)** Correlation analysis between soil bacterial wilt pathogen content and soil microorganisms. Correlations with |r| ≥ 0.8 and p< 0.05 are displayed. (Spearman). The width of chord connections represents the magnitude of the absolute correlation value, with red indicating positive correlation and blue indicating negative correlation. **(D)** Correlation analysis between soil phenolic acid and microorganisms. **(E)** Correlation between soil differential metabolites and phenolic acids. **(F)** Correlation between soil differential metabolites and microorganisms.Data are means of six replicates. Red indicates positive correlation, Blue indicates negative correlation; Thinner ellipses represent larger absolute correlation values, Darker colors represent larger absolute correlation values; (*p < 0.05, **p < 0.01, ***p<0.001, Spearman).

The association between tobacco bacterial wilt and differential soil metabolites, phenolic acids, and the microbiome is shown in **Figure 8**. As shown in **Figure 8A**, tobacco bacterial wilt was significantly positively correlated with differential metabolites such as acetoacetic acid and significantly negatively correlated with quinolinic acid. **Figure 8B** shows that bacterial wilt was significantly positively correlated with phenolic acids such as quinic acid, p-coumaric acid, o-coumaric acid, catechol, chlorogenic acid, and rutin. Correlation analysis between soil-borne bacterial wilt content and microorganisms showed a significantly positive correlation with Mucoromycota, Bacteroidota, Pseudomonadota, *Marmoricola*, *Nocardioides*, and *Rhodanobacter* (**Figure 8C**).

As shown in **Figures D–F**, the differentially abundant metabolites rhamnose, D-allose, and mannitol were significantly negatively correlated with syringic acid, ferulic acid, caffeic acid, gallic acid, phthalic acid, and protocatechuic acid. A significant negative correlation for differentially abundant metabolites was also noted for Chloroflexota, Gemmatimonadota, Acidobacteriota, *Pseudolabrys*, *Bradyrhizobium*, and *Sphingomonas*, as opposed to significant positive correlations with *Nocardioides*, *Rhodanobacter*, and *Marmoricola*. Syringic acid, ferulic acid, caffeic acid, gallic acid, phthalic acid, and protocatechuic acid, among other phenolic acids, are significantly positively correlated with microorganisms such as Chloroflexota, Gemmatimonadota, Acidobacteriota, *Pseudolabrys*, *Bradyrhizobium*, and *Sphingomonas*.

## 4 Discussion

### 4.1 Soil pathogens and phenolic acid content in tobacco-sweet potato intercropping

Intercropping can enhance the stress tolerance of crop populations and control or mitigate the occurrence of certain pests and diseases (Jia et al., 2016; Zheng et al., 2023, 2023). This study found that, compared to the T treatment, the TSP treatment not only reduced the incidence of bacterial wilt in flue-cured tobacco but also significantly decreased the content of *Ralstonia solanacearumin* tobacco-growing soil by 21.4%, indicating that intercropping can enhance resistance to bacterial wilt in flue-cured tobacco. Phenolic acids are allelochemicals in continuous cropping soils, exerting inhibitory or promotional effects on crop growth (Xie et al., 2014), and influencing soil microbial community structure (Liu et al., 2020). The results of this study revealed that the phenolic acid content in the TSP treatment was significantly higher than that in the T treatment. Tobacco roots secrete phenolic acids such as ferulic acid, cinnamic acid, benzoic acid, vanillic acid, and p-hydroxybenzoic acid (Zhang et al., 2013), while sweet potato roots contain phenolic compounds including chlorogenic acid, caffeic acid, and 3-caffeoylquinic acid (Musilová et al., 2017). Compared to monoculture, the presence of an additional crop in intercropping systems inevitably leads to significant differences in the types, quantities, and composition of root exudates (Zhu et al., 2017). It can thus be inferred that the phenolic acids in TSP soil are derived from both crops or result from root interactions between the two species.

The changes in microbial community structure induced by phenolic acids are attributed to their selective disruption of the bacterial-to-fungal ratio, leading to increased pathogenic fungal biomass, accumulation of pathogenic microorganisms, and reduced microbial diversity (Qu and Wang, 2008). For example, in the optimal biochar application treatment for controlling tobacco bacterial wilt, the highest disease suppression efficacy did not correspond to the highest of soil microbial diversity (Wang, Wang et al., 2024). In our study, both bacterial wilt pathogen content and soil microbial diversity decreased under the TSP system. This suggests that the reduction in *Ralstonia solanacearum* content in the TSP treatment is not achieved by increasing soil microbial diversity or enhancing interspecific competition. One explanation may be that the TSP treatment increases phenolic compound content in the soil, leading to a high-concentration phenolic inhibition effect that simultaneously reduces both *Ralstonia solanacearum* content and soil microbial diversity.

### 4.2 Differential carbohydrate metabolites in tobacco-sweet potato intercropped soils

Sugar metabolites not only provide energy for soil microorganisms but also interact with immune signaling molecules to participate in plant immunity (Long et al., 2003). The sugar content in corn plants during the grain-filling stage is significantly correlated with resistance to corn stalk rot (Luo and Gao, 1997). After inoculation with pathogenic bacteria, sugar content in cucumber leaves increases, enhancing resistance to downy mildew (Li et al., 2005). Elevated sugar accumulation in kiwifruit branches and leaves exhibits a significant inhibitory effect on plant pathogens (Yuan, Yuan et al., 2023). Specific sugars exhibit direct antimicrobial or resistance-inducing effects; L-rhamnose significantly inhibits tomato bacterial wilt (Wang, 2021), while D-allose can enhance systemic resistance to bacterial wilt (Zhang et al., 2020). Interestingly, sugars can also be exploited by pathogens. Ralstonia solanacearum, for example, utilizes sucrose, sorbitol, and mannitol as carbon sources to support its growth (Wang, 2008). Conversely, mannitol functions as an osmoprotectant synthesized by plants under biotic stress and plays a key protective role in fungal resistance (Zhao et al., 2019).

Thus, sugar compounds exert a bidirectional regulatory effect on soil microorganisms: they serve as a carbon source for microorganisms and drive rhizosphere metabolism, while also participating in plant immunity through concentration-dependent mechanisms to inhibit microorganisms. In this study, the rhizosphere soil of the T treatment exhibited significantly higher levels of the differentially abundant metabolites rhamnose, mannitol, and D-allose compared to the TSP treatment, and these were significantly correlated with the content of bacterial wilt pathogen content. However, the underlying causes and mechanisms of this phenomenon remain unclear.

### 4.3 Microbial community structure in the rhizosphere soil of tobacco-sweet potato intercropping

The effects of different intercropping combinations on soil microbial diversity vary (Correa-Galeote et al., 2016; Ablimit et al., 2022; Zhang et al., 2022). For example, maize-soybean intercropping exhibits higher microbial diversity compared to monoculture (Jiang et al., 2024), whereas maize-peanut intercropping shows no significant impact on microbial diversity or community structure, with only minor differences in the abundance of a few bacterial and fungal phyla (Zhao et al., 2022). This suggests that bacterial and fungal diversity may depend more on the identity of adjacent crops rather than the cropping pattern itself. Secondary metabolites secreted by the roots of intercropped plants, such as organic acids, sugars, and phenolic acids, may negatively affect microbial species and abundance (Li et al., 2022). In this study, both microbial diversity and richness were lower in the TSP treatment than in the T treatment. Thus, the reduction in microbial diversity in the intercropping system may be related to the root activities of tobacco and sweet potato (Zhao et al., 2022).

In the TSP treatment, the abundances of Chloroflexota, Acidobacteriota, *Streptomyces*, and *Sphingomonas* were enriched, while those of Pseudomonadota, Actinomycetota, Bacillota, *Nocardioides*, and *Rhodanobacter* were depleted—a conclusion aligned with findings of Zhang et al (Zhang et al., 2025). Acidobacteriota plays an important role in soil material cycling and ecological environment construction (Liu et al., 2016). *Sphingomonas* is one of the most effective microorganisms for degrading toxic substances in soil and can promote nutrient absorption in the rhizosphere and resist various pathogens (Liu et al., 2023). Streptomyc*es* can exert antagonistic effects against pathogens through the production of bioactive compounds (Sun et al., 2025). This may be due to the reduction of beneficial microorganisms in tobacco monoculture, leading to an increase in pathogenic microorganisms. *Bradyrhizobium* can inhibit pathogens (Imran A. Siddiqui, 2002); *Rhodanobacter* have been demonstrated to exhibit antagonistic effects against the root rot fungal pathogen *Fusarium solani* (Zhong et al., 2022); and *Nocardioides* possesses antifungal activity, enabling it to control plant pathogenic fungi (El-Sayed et al., 2023). However, this study found that the abundances of *Bradyrhizobium*, *Nocardioides*, and *Rhodanobacter*were lower in TSP than in T, suggesting that changes in these microorganisms may not contribute significantly to the suppression of *Ralstonia solanacearumin* flue-cured tobacco.

Consequently, the increased abundances of Chloroflexota, Acidobacteriota, Streptomyces and Sphingomonas, coupled with decreased abundances of Pseudomonadota, Actinomycetota, Bacillota, Rhodanobacter, Nocardioides, and Marmoricola, provide substantial evidence elucidating the decline in Ralstonia solanacearum within tobacco-sweet potato intercropping systems.

### 4.4 Relationship between Ralstonia solanacearum content and soil phenolic acids, metabolites, and microorganisms

Root exudates provide carbon sources for plants and soil microorganisms (Joelle et al., 2018) and can recruit microorganisms in the root zone. In this study, the content of *Ralstonia solanacearum* was positively correlated with sugars such as rhamnose, D-allose, and mannitol, indicating that these sugars may serve as important carbon sources and energy substrates for pathogen growth. Phenolic compounds play a key role in plant defense against reactive oxygen species (ROS) and have inhibitory effects on plant pathogens (Vidot et al., 2020). For example, root exudates such as caffeic acid, protocatechuic aldehyde, and gallic acid significantly inhibit *Ralstonia solanacearum* (Gu et al., 2016; Sowndarya et al., 2020; Yu et al., 2022). The interactions between phenolic acids and soil microorganisms are complex and bidirectional. Qu et al. demonstrated that phenolic acids can significantly influence microbial biomass, diversity, and community structure in soil, selectively enhancing specific microbial species (Qu and Wang, 2008). Microorganisms can also degrade phenolic acids; for instance, *Pseudomonas* and *Sphingomonas* exhibit efficient degradation capabilities for benzoic acid and *p-*hydroxybenzoic acid (Zhang et al., 2024a, 2024). An increase in *Ralstonia*(a genus within Pseudomonadota) (She and He, 2020)and a decrease in *Sphingomonas* abundance (Fan et al., 2023) are key factors contributing to the aggravated severity of tobacco bacterial wilt. This study found that the content of *Ralstonia solanacearum* was negatively correlated with phenolic compounds (e.g., syringic acid, ferulic acid, gallic acid) and beneficial rhizobacterial communities (e.g., Chloroflexota, Acidobacteriota, *Sphingomonas*, *Pseudomonas*). Based on the above, since beneficial microorganisms can degrade phenolic acids, and both phenolic acids and microorganisms can suppress disease, does this imply a contradiction between the two? We hypothesize that this is because phenolic acids originate from diverse sources, and thus the degradation of phenolic acids by microorganisms may play a relatively minor role.

**Figure 9:**
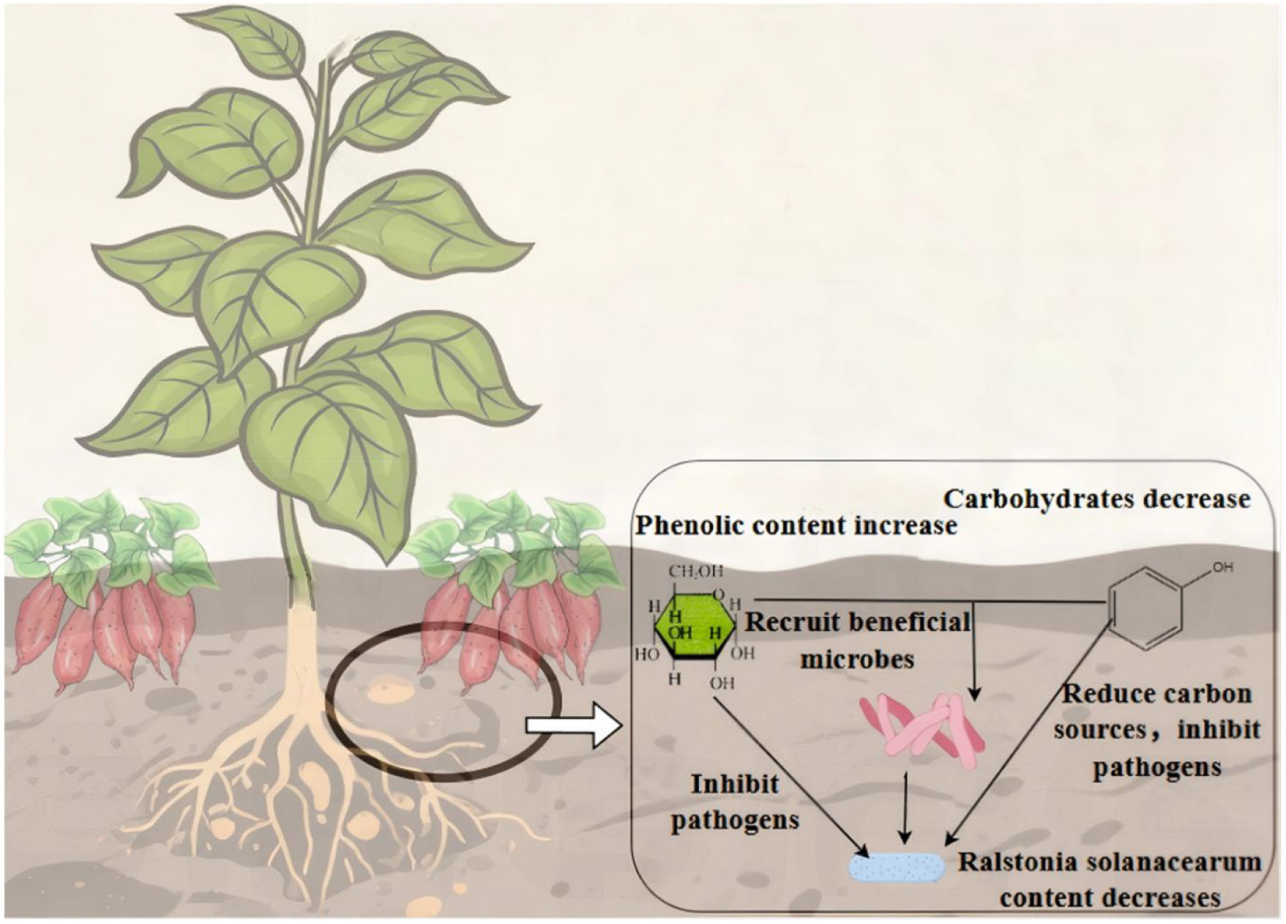
Schematic diagram of the synergistic inhibitory effect of soil phenolic acids, metabolites, and microorganisms on *Ralstonia solanacearum.*.

In summary (**Figure 9**), we hypothesize that TSP treatment increases phenolic acid content while downregulating sugar compounds relative to T treatment. Sugars function bidirectionally: as carbon sources driving pathogen proliferation and as immune signaling molecules participating in plant immunity. Under intercropping conditions with reduced sugar levels, pathogen utilization of carbon sources is limited, thereby partially inhibiting pathogen growth. Phenolic acids directly suppress pathogen development, while the combined changes in sugar and phenolic acid levels increase the abundance of beneficial microorganisms that collectively resist pathogens. Consequently, phenolic acids and beneficial microbes may form a multi-dimensional synergistic disease resistance network, jointly contributing to the suppression of bacterial wilt.

Although this study identified the impacts of TSP treatment on soil phenolic acids, microbial communities, and metabolomics alongside its significant correlation with reduced bacterial wilt incidence, the underlying mechanisms warrant further investigation. Specifically, how TSP intercropping induces beneficial metabolite accumulation and enriches protective microbial consortia, as well as the pathways through which it suppresses *Ralstonia solanacearum* proliferation and pathogen-facilitating microbiota, necessitates thorough investigation. Therefore, elucidating the mechanisms through which TSP rhizosphere bacteria and differential soil metabolites inhibit pathogen colonization, growth, and disease development represent a critical future research priority.

## 5 Conclusion

This study found that intercropping tobacco with sweet potatoes significantly reduced the incidence of bacterial wilt in flue-cured tobacco and decreased the content of *Ralstonia solanacearumin* the soil by 21.4%, while increasing the total phenolic content in the soil by 21.9%. After intercropping, differentially expressed metabolites in the rhizosphere soil were significantly enriched in carbohydrate metabolism pathways such as nucleotide sugar biosynthesis, fructose, and mannose metabolism. The main downregulated metabolites included rhamnose, D-allose, and mannitol, in parallel to the accumulation of certain phenolic acids, such as syringic acid, ferulic acid, caffeic acid, and gallic acid. This led to an increase in the abundance of beneficial microorganisms such as Chloroflexota, Gemmatimonadota, Acidobacteriota, and *Sphingomonas* in the rhizosphere soil. These changes in substance concentrations are closely associated with a decrease in the content of the bacterial wilt pathogen in tobacco-sweet potato intercropping soil. Tobacco-sweet potato intercropping may significantly reduce the bacterial wilt pathogen in tobacco by regulating the metabolic-microbial interaction balance in the soil, thereby helping to reduce the risk of disease occurrence. Collectively, this study provides practical guidance for designing sustainable flue-cured tobacco production systems, establishes a foundation for developing scalable intercropping techniques, and offers a theoretical basis for leveraging intercropping to regulate soil microecology and enhance crop disease resistance.

## Data Availability

The original contributions presented in the study are publicly available. This data can be found here: NCBI, PRJNA1353649.
